# Functional Roles of E6 and E7 Oncoproteins in HPV-Induced Malignancies at Diverse Anatomical Sites

**DOI:** 10.3390/cancers8100095

**Published:** 2016-10-19

**Authors:** Vjekoslav Tomaić

**Affiliations:** 1 International Centre for Genetic Engineering and Biotechnology, Padriciano 99, I-34149 Trieste, Italy; tomaic@irb.hr; 2 Division of Molecular Medicine, Rudjer Boskovic Institute, 10000 Zagreb, Croatia

**Keywords:** HPV, cancer, E6 and E7 oncoproteins

## Abstract

Approximately 200 human papillomaviruses (HPVs) infect human epithelial cells, of which the alpha and beta types have been the most extensively studied. Alpha HPV types mainly infect mucosal epithelia and a small group of these causes over 600,000 cancers per year worldwide at various anatomical sites, especially anogenital and head-and-neck cancers. Of these the most important is cervical cancer, which is the leading cause of cancer-related death in women in many parts of the world. Beta HPV types infect cutaneous epithelia and may contribute towards the initiation of non-melanoma skin cancers. HPVs encode two oncoproteins, E6 and E7, which are directly responsible for the development of HPV-induced carcinogenesis. They do this cooperatively by targeting diverse cellular pathways involved in the regulation of cell cycle control, of apoptosis and of cell polarity control networks. In this review, the biological consequences of papillomavirus targeting of various cellular substrates at diverse anatomical sites in the development of HPV-induced malignancies are highlighted.

## 1. Introduction

Persistent infection with the human papillomaviruses (HPVs) is the main risk factor in the development of numerous human malignancies at various anatomical sites [1,2]. Of these, cervical cancer is the most important disease, predominantly affecting women in developing countries and causing more than 600,000 cancers annually [2,3]. Although around 200 HPV types are known to infect humans, only a small proportion of these have been associated with cancer development [4,5]. HPVs are classified into five genera: alpha (α), beta (β), gamma (γ), mu (μ) and nu (ν), and the α and β genera have been most intensively investigated [6]. The α-papillomavirus group contains members which infect mucosal epithelia and which are divided into low-risk (LR) and high-risk (HR) types, based on their ability to cause cancer. The LR viral infections result in benign lesions, commonly caused by HPV-6 and HPV-11, while HR viral infections have been associated with malignancies of anogenital and head-and-neck regions. Of these, HPV-16 and HPV-18 cause approximately 80% of the world’s cervical cancer burden, while the remaining 20% are predominantly associated with other HR HPVs such as HPV-31, HPV-33, HPV-45 and HPV-58 [7,8]. Interestingly, in HPV-positive head-and-neck cancers, which primarily affect the oropharynx and occur in the tonsils and base of the tongue, HPV-16 is most prevalent, while the other HR types are only rarely detected [9,10].

β-HPVs commonly infect cutaneous epithelia. Originally they were found to be present in skin warts and in cutaneous squamous cell carcinomas (CSCC) of patients with the rare disease epidermodysplasia verruciformis (EV) [11]. In EV patients, HPV-5 and HPV-8 have been characterized as potentially cancerogenic types [4,5]. CSCC development usually occurs a few decades after the initial formation of benign skin lesions, predominantly in sun-exposed areas, indicating that the primary infections occur early in life [12]. It had been thought that the mechanism of action of β-HPVs in the development of skin cancer was equivalent to the mechanism of HR HPVs in cervical cancer development. However, β-HPV genomes do not integrate in the host DNA [13], and there is no evidence of continuous oncoprotein expression in CSCC, which suggests that β-HPVs may play a role in tumour initiation, but not in tumour maintenance [11,14]. Furthermore, cellular transformation and mouse model studies have indicated that, in the presence of UV damage, E6 and E7 from β-HPVs can contribute towards initiation of cancer formation [15,16,17].

## 2. Viral Life Cycle Differences between Alpha and Beta-HPV Types

**Figure 1:**
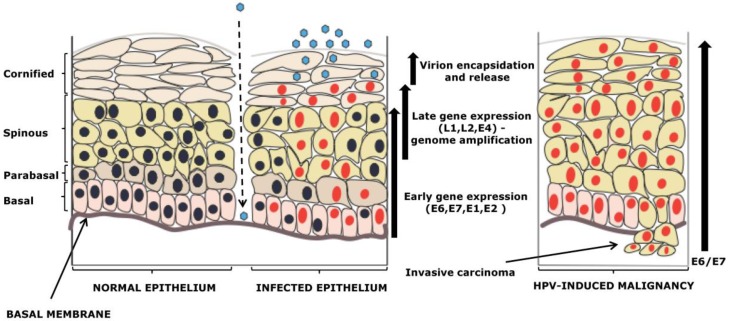
From a productive HPV life cycle to cancer development. The left panel shows the proposed route of HPV infection via microinjuries in mucosal epithelium. There is a highly controlled expression of several viral gene products, where E6/E7 oncogenes, by targeting their respective substrates p53/pRb, promote continual cell proliferation. This allows the virus to amplify its genome, complete its productive life cycle and ultimately produce new virions. If the immune system of the host does not resolve the viral infection and it persists for a long period of time, this may result in HPV-induced malignancy, in which the virus fails to complete its life cycle, while the E6 and E7 proteins are highly overexpressed. Adapted from [8].

Alpha-HPV types infect mucosal epithelia and, as demonstrated in Figure 1, it is believed that the virus enters through microinjuries and infects the basal cells of the epithelium. The viral life cycle is strictly dependent upon the cellular differentiation of keratinocytes, the principal target cells. The virus does not have its own replicative machinery and is therefore dependent upon cellular division and stratification of the epithelium, which occurs from the basal layers towards the suprabasal layers, exploiting this process to replicate and produce new viruses. In this process HPV oncoproteins, E6 and E7, play a crucial role. Their joint action targeting of diverse cellular pathways involved in the regulation of cell cycle control and apoptosis enables the virus to maintain cell proliferation in highly differentiated suprabasal regions, allowing viral genome amplification to occur [1,18].

Much information about the viral life cycle has been obtained from the organotypic raft culture model system, which reconstructs the full productive life cycle of HPVs in vitro [19]. In this system either the spontaneously immortalized keratinocyte cell line Normal Immortal Keratinocytes (NIKS) or Human Foreskin Keratinocytes (HFKs) immortalized by telomerase are used to recapitulate the replicative viral life cycle [20]. NIKS are very effective in supporting the life cycle of a number of HR α-types such as HPV-16, -18, -31, -45 and -58, while the immortalized HFKs have been used to study the life cycle of some of the LR types such as HPV-11 [21,22,23]. However, there are some limitations to this model of studying HR HPV viral life cycles, since all the analyses are performed in the same cellular background. It is very well known that HPVs infect various anatomical sites, which have different epithelial backgrounds and there is a high possibility there could be significant differences in the viral life cycle depending on the cell origin. Unfortunately, owing to these limitations there is currently no solid information about the viral life cycle at other sites of infection, although studies have reported the detection of HPV-16 L1 protein in virus-infected areas such as tonsils, indicating the ability of the virus to complete its productive life cycles in the oropharyngeal region [24]. Therefore, there is a pressing need to extend existing organotypic raft culture systems to cells isolated from other anatomical sites to better understand potential differences in the productive life cycle of the virus.

There have been few successful attempts to study β-HPVs life cycles; although some limited information has been obtained from organotypic raft culture studies that have shown certain similarities between LR α types and β types. Like HPV-11, HPV-8 episomal genomes are not well maintained in tissue culture and tend to be lost over time. In addition, it appears that HPV-8 viral genomes do not increase the cellular growth rate, which is why the complete HPV-8 life cycle has not been reproduced in this in vitro system [14,25]. This could be partly attributed to β-HPV types not expressing an E5 protein, which in α-HPVs can contribute to genome amplification by stabilizing the EGFR [16,26,27,28]. In addition, HPV E5 protein also has additional functions that contribute to optimal life cycle completion, for example, HR HPV-16 E5 inhibits the EVER/ZnT-1 protein complex, which is involved in maintaining the cellular zinc balance which plays a role in controlling HPV infections [29]. Since the β-HPV types do not encode E5 they cannot inhibit EVER proteins in the same way. However, mutations in either of two genes (EVER1 and EVER2) were shown to be causative or contributive agents in most EV cases, and might thereby contribute indirectly to β-HPV infections. This might partially explain the host restrictions for infections with β-HPV types [29,30].

## 3. The Role of the High Risk Alpha E6 and E7 in Pathogenesis

**Figure 2:**
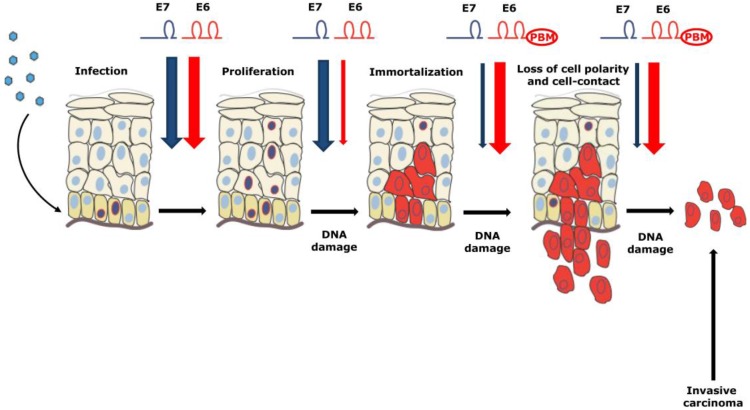
The joint action of HPV E6 and E7 oncoproteins is required for HPV-induced malignancy. By targeting pRb E7 promotes tumor formation and contributes to the early stages of HPV-driven malignancy. It is believed that the later stages of malignancy are in part driven by E6 and its ability to target the PDZ-domain containing cellular substrates via its C-terminal PDZ-binding motif (PBM). Adapted from [45].

The normal productive viral life cycle of the HR α-types is a highly regulated and coordinated process. However, in some instances, mostly during persistent infection, the viral DNA is randomly integrated into the host genome, leading to cellular immortalization and eventually to malignant progression. The outcome of this process is the collapse of the viral replicative capacity: most of the viral genes are lost while the two major viral oncogenes, E6 and E7, remain uncontrollably expressed, driving cellular immortalization, further progressing towards cellular transformation, and ultimately resulting in cancer development [31,32]. Interestingly, the process of viral integration occurs much less frequently in oropharyngeal carcinomas than in cervical cancer [33]. The importance of E6 and E7 in maintaining the transformed phenotype can be seen from their continual expression in tumors and derived cell lines many years after the primary immortalizing events. This was observed in numerous studies which demonstrated that any inference with these oncogenes at either the RNA (ribozymes, anti-sense RNA and RNA interference) or protein levels (blocking peptides) will lead to cell growth arrest and/or apoptosis [34,35,36,37,38,39]. Moreover, it has been shown that the complementary activities of E6 and E7 are required to immortalize primary human keratinocytes; E6 interfering with cell survival pathways and E7 promoting cellular proliferation [40,41,42]. In contrast, neither E6 nor E7, when expressed alone, had any significant impact on cellular immortalization. Transgenic mouse model cervical cancer studies showed that E7 increased proliferation and centrosome copy number and induced the progression of multifocal microinvasive cervical cancers; while E6 elevated centrosome copy number and eliminated detectable p53 protein, but did not produce neoplasia or cancer. Importantly, the combination of both oncoproteins resulted in increased centrosome number and large, extensively invasive, cancers [43]. Similar observations were seen in models of head-and-neck squamous cell cancers (HNSCC). In these, E7 was shown to be the major transforming oncoprotein, whereas E6 appears to be more likely to play a secondary role in contributing to the later stages of malignancy [44]. Based on these studies, a model of HPV-induced carcinogenesis has been developed and is summarized in Figure 2. Thus, these two oncoproteins are considered to be excellent targets for therapeutic intervention, and understanding the molecular mechanisms underlying their respective functions is critical for developing such antiviral therapies.

### 3.1. Alpha-HPV E6

#### 3.1.1. p53 and Components of the Proteasome Ubiquitin Pathway as Interacting Partners of Alpha-HPV E6

**Figure 3:**
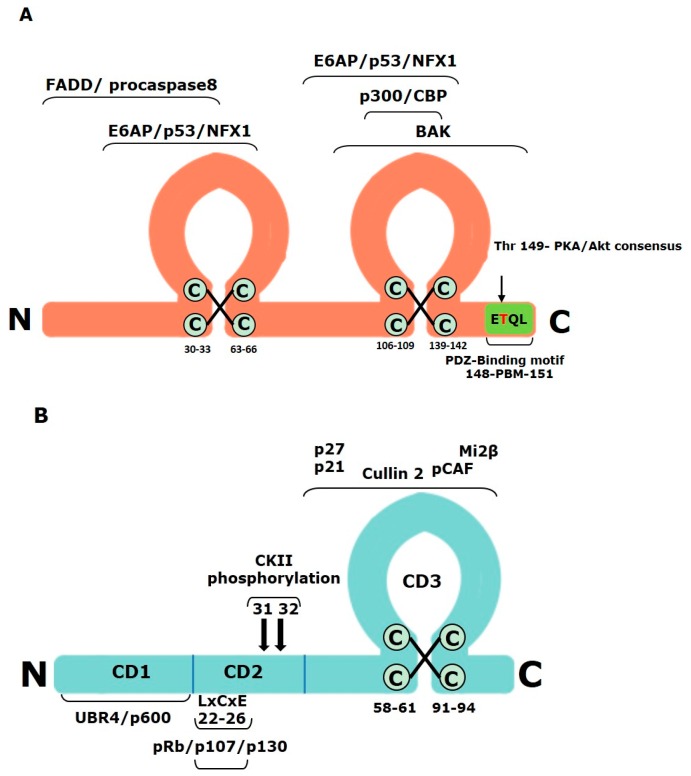
The high-risk HPV E6 and E7 oncoproteins. (**A**) Schematic diagram of HPV-16 E6 showing the position of amino acid motifs that are important for protein integrity and function. The two zinc fingers are shown, together with regions that are involved in interacting with some of its cellular target proteins. The C-terminal PBM is shown and the PKA phosphorylation site is also indicated; (**B**) Schematic diagram of E7 and the most important amino acid motifs required for integrity and protein functions. Three regions of E7 that are homologous to adenovirus E1A conserved regions 1-3 (CD1-3) are shown. The zinc finger is also shown, together with the regions involved in pRb binding (LXCXE) and the two serine residues (31 and 32) that are susceptible to casein kinase II (CKII) phosphorylation.

The HPV E6 protein is approximately 150 amino acids long and has two zinc fingers formed by two pairs of CXXC motifs [46,47] (Figure 3A). These motifs are strictly conserved in all E6 proteins and their integrity is essential for the oncoprotein’s normal functions [48,49]. The crystal structures of both N-terminal and C-terminal halves, as well as the complete structure of the E6 proteins have been resolved [50,51,52]. These structural studies have additionally confirmed the fact that E6 interacts with a wide range of cellular substrates [53]. The principal cellular target of HR E6 proteins is the tumor suppressor p53. In the absence of HPV, p53 is regulated by the RING finger domain-containing ubiquitin ligase Mdm2 [54]. However, under stress, such as upon DNA damage or viral infection, this process is abolished and p53 is both stabilized and activated by a series of phosphorylation events [55]. In HPV-positive cancer cells, the Mdm2 pathway is completely inactive and p53 turnover is regulated by HPV E6 [56]. In addition to p53 protein regulation, which can be proteasome-dependent or -independent, α-HPV E6s are also involved in regulation of p53 gene transactivation. It was shown that E6 mutants unable to induce p53 degradation can still abolish p53 transcriptional transactivation activity [57]. E6 can also control p53-dependent gene regulation, via its interactions with p300/CBP co-activators [58,59]. Additionally, it was demonstrated that E6-interacting regions of p300 are necessary for E6 to inhibit p53-dependent chromatin transcription, and that the E6-mediated repression of p53-dependent activation correlates with inhibition of acetylation on p53 and nucleosomal core histones, altering p53 and p300 recruitment to chromatin. This process is a unique way in which E6 represses p53 which does not require the proteasome [60].

On its own, E6 cannot target p53 for proteasomal degradation, rather, it hijacks a cellular E3 ubiquitin ligase UBE3A/E6AP (E6-associated protein), binding through E6’s LXXLL motif, and the stable E6/E6AP complex then labels p53 for degradation in a proteasome-dependent manner [61,62,63,64]. Recent studies have further emphasized the importance of the E6/E6AP association, showing that the stability of HR α-HPV E6 proteins is critically dependent upon the presence of E6AP [65]. A number of studies have shown E6 to be closely associated with other components of the proteasome degradatory pathway: in addition to E6AP, HR α-HPV E6 interacts with the E3 ubiquitin ligases UBR5/EDD and HERC2 [66,67]. It has been shown that changes in the UBR5/EDD protein levels throughout the viral life cycle, or during malignancy, might affect E6’s ability to target its cellular substrates for proteasomal degradation, and thus might have a direct impact on the development of HPV-induced malignancy [66]. In addition, HR α-HPV E6s interact directly with the S5a proteasome subunit, one of the two major ubiquitin acceptor molecules of the proteasome. The interaction is E6AP-dependent and results in the upregulated ubiquitination of S5a, indicating the complexity of E6 association with the proteasome [68].

#### 3.1.2. Alpha-HPV E6 and Apoptosis

Although the major anti-apoptotic activity of E6 is its inactivation of p53, it also has a number of other cellular pro-apoptotic targets, including Bak. Bak is highly expressed in the upper epithelial layers, indicating that it could play a role in terminal differentiation [69]. After UV exposure it is activated and stabilized independently of p53 [70] and HPV E6 targets Bak for E6AP-dependent proteasome-mediated degradation, thereby directly inhibiting apoptosis [15,71,72]. LR HPV types, such as HPV-11, also target Bak for proteasome-mediated degradation, however their capacity to degrade Bak is fairly weak, which correlates with a reduced anti-apoptotic activity of the LR mucosal types [72]. In addition to Bak, other cellular proteins involved in apoptosis regulation have been reported as E6 targets: survivin was identified as an indirect target of HPV E6, which strongly upregulates survivin promoter activity, again resulting in the suppression of apoptosis [73]. Furthermore, E6 also interacts with other components of the host apoptotic machinery, such as the tumor necrosis factor receptor 1 (TNF R1) [74], the adaptor molecule Fas-associated death domain (FADD) [75], and procaspase 8 [76]. E6 interacts with procaspase 8 and FADD through their death effector domains (DEDs) and mediates the accelerated degradation of both proteins.

#### 3.1.3. Alpha-HPV E6 and DNA Replication

In addition to their association with pro-apoptotic cellular proteins, HR E6 oncoproteins are also involved in the deregulation of the cellular DNA replication machinery. Normal somatic cells end their life by entering senescence, which results from the progressive shortening of telomere DNA over successive rounds of replication, and most normal cells do not exhibit telomerase activity [77]. However, activated telomerase is found in cells of cervical carcinomas and high-grade lesions induced by HR HPVs, preventing shortening of the telomeres and leading to continued proliferation [78]. HPV-16 E6 induces telomerase activity in primary epithelial cells through transcriptional transactivation of the hTERT telomerase catalytic subunit [79,80], which probably requires the presence of an intact E box in the minimal hTERT promoter [80,81,82]. HPV-16 E6 hTERT activation was shown to require E6 binding to Myc [81,83], but not to E6AP [84]. On the other hand, E6/E6AP-dependent degradation of the transcriptional repressor NFX1-91, which binds to the hTERT promoter, causes the mSin3A/HDAC complex to dissociate from the hTERT promoter, inducing hTERT transcription [85]. Thus, although the exact mechanism of activation seems to be controversial, hTERT activation by E6 appears to be a crucial step for HPV-induced malignancy.

#### 3.1.4. Alpha-HPV E6 and PDZ Domain-Containing Proteins as Interacting Partners

All HR α-HPV E6 proteins have a class I PDZ (PSD95/Dlg/ZO-1)-binding motif (x-T/S-x-L/V [86]) at their C-termini. PDZ domains are approximately 90 amino acid protein-protein interaction domains [87] and PDZ domain-containing proteins fall into three main groups: PDZ-only proteins; Membrane associated guanylate kinases (MAGUKs); and PDZ proteins with other binding domains [88]. PDZ domain-containing proteins typically function as scaffolds that assemble signaling complexes and localize them to specialized regions of cell-cell contact, such as Adherens and Tight Junctions [87]. The two major HR types, HPV-16 and HPV-18, are known to interact with numerous PDZ-domain containing proteins via their PDZ-binding motifs (PBMs) [8,45]. Several of these are involved in the regulation of epithelial cellular polarity, emphasizing the importance of this pathway for both viral replication and for HPV-driven malignancy, as has been shown in both tissue culture [89,90] and transgenic animal model systems [91]. The PBM is also important in the viral life cycle, since its loss in the context of an intact viral genome reduces viral replicative potential and very often leads to episomal integration [92,93].

The specificity of the PDZ interaction is an important factor in E6-PDZ target recognition, and different E6 proteins interact preferentially with different PDZ domain-containing proteins [1,8,45,94]. For example, HPV-18 E6 binds more strongly than HPV-16 E6 to Dlg1 and MAGI-1 [89], and exchanging the last amino-acid residue of HPV-16 E6 (L) for that of HPV-18 E6 (V) reverses this phenotype [95]. Conversely, HPV-16 binds hScrib more strongly than HPV-18 E6 and this is also PBM sequence-dependent [96]. Interestingly, several of E6’s PDZ domain-containing targets are potential tumor suppressors, with hScrib being an excellent candidate for a tumor suppressor in diverse cancer settings [97,98]. The importance of the exact PBM sequence in substrate recognition was supported by crystal structure studies which showed that a peptide homologous to the HPV-18 E6 carboxy-terminus binds differently to different PDZ domains [99,100]. Interestingly, Rhesus papillomavirus 1 (RhPV 1), a HR mucosal type whose E6 and E7 oncogenes share approximately 50% homology with those of HPV-16, has no C-terminal PBM on E6; instead a PBM is found on the E7 C-terminus. This is also a class I PBM, but with a significantly different sequence from those of HPV-16 and HPV-18 [8,101]. Correspondingly, the preferred target of the RhPV-1 E7 PBM also differs, and is the cell polarity regulator Par3, which it targets in part for proteasome-mediated degradation [101]. This demonstrates a remarkable evolutionary conservation of this activity between HR virus types—to target the same cell polarity control pathway by similar means, but through different proteins.

#### 3.1.5. Phospho-Regulation of Alpha-HPV E6 PDZ-Binding Motif

Interestingly, the HR PBM can also be post-translationally regulated. It contains a protein kinase A (PKA) phospho-acceptor site, and its phosphorylation by either PKA or AKT blocks PDZ binding and instead allows E6 to interact with 14-3-3ζ [102], a member of the 14-3-3 family of phospho-threonine/serine interacting proteins [103], which are involved in the regulation of a number of cellular processes directly relevant for cancer progression and malignant development. The intact PBM is necessary for interactions between E6 and 14-3-3ζ, and downregulation of 14-3-3ζ leads to a dramatic decrease of E6 protein levels in HPV-18 positive HeLa cells [102]. It was also shown that the phosphorylation-dependent switch between PDZ- or 14-3-3ζ-binding activity is dependent upon the different susceptibilities of the individual HPV types to PKA or AKT phosphorylation of the PBM. This regulation was shown to be well-conserved between HPV-16, HPV-18, and HPV-58 E6s, but somewhat weaker between HPV-31, HPV-33, and HPV-51 E6s [104]. All of this highlights the importance of the PBM and its role in HPV-induced carcinogenesis.

### 3.2. Alpha-HPV E7

HPV E7 is a 98 amino acid protein that has a C-terminal zinc-binding domain, whose structural integrity is critical for E7 activity [105,106]. The protein contains three conserved domains, CD1, CD2, and CD3 (Figure 3B): CD1 and CD2 correspond to small parts of conserved regions 1 and 2 (CR1 and CR2) of Adenovirus E1a, while CD3 is homologous to SV40 large T antigen. The overall integrity of the protein is critical for optimal E7 interactions with its cellular substrates, although most of the characterized functions of E7 have been mapped to the CD2 and CD3 regions [107] (Figure 3B).

E7 is post-transcriptionally regulated by the proteasome and by phosphorylation. It interacts with the Skp-Cullin-F box (SCF) ubiquitin ligase complex, which leads to increased ubiquitination of the protein [108]. It has been also demonstrated that Casein Kinase II (CKII) phosphorylation of the E7 N-terminal domain is critical for its transformational activity [109,110]. An additional C-terminal phosphorylation site has been identified which seems to be phosphorylated primarily during the S phase, but the kinase responsible has not been determined [111].

#### 3.2.1. Conserved Domains of Alpha-HPV E7

The CD1 domain comprises the first 20 amino acids of E7 and is critical for E7’s ability to induce S-phase progression and cellular transformation [112,113,114] (Figure 3B). Among the binding partners that interact with this domain of E7 are UBR4/p600 [115] and p300/CBP-associated factor (P/CAF) [116]. UBR4/p600 is essential for membrane morphogenesis, which is crucial for cell migration, and is also required for cell survival [117] and anchorage-independent growth in both HPV-negative and HPV-positive cells. Interaction between E7 and UBR4/p600 is thought to be required for E7-mediated cell transformation [118], but its exact function in E7’s activities still remains to be determined. More recent studies have shown that E7 proteins from many HPV types, including those that are not cancer-associated, interact with UBR4/p600; and it has been speculated that UBR4/p600 could play a role in viral replication [119]. On the other hand, the consequences of the E7 and P/CAF interaction appear to be more straightforward, since E7 has been shown to down-regulate the P/CAF mediated activation of NF-κβ family members, which occurs during viral infection, resulting in viral escape from the immune response [116].

Amino acid residues from 20 to 38 constitute the CD2 region of E7 (Figure 3B). This contains the CKII phosphorylation site and the LXCXE binding motif involved in binding to proteins such as the retinoblastoma tumor suppressor (pRb). As mentioned above, the CKII phospho-acceptor site is important for E7’s transforming capacity [110,120] and for its ability to drive S-phase progression [121], while interaction with the pocket proteins has been characterized as one of the major functions of E7.

pRb has a critical role in cell cycle regulation, controlling the G1 to S phase transition. Under normal conditions, pRb is unphosphorylated in early G1 and is progressively phosphorylated towards S phase. Unphosphorylated pRb interacts with the E2F transcriptional factors and acts as a transcriptional repressor of promoters containing E2F sites [122]. E2F family members are involved in the cell cycle-dependent transcriptional regulation of many genes involved in DNA synthesis [123]. In the HPV-positive cell, E7 uses its LXCXE motif to target unphosphorylated pRb for degradation [124] via the ubiquitin proteasome pathway [125], and HPV-16 E7’s association with the cullin 2 complex contributes to this process [126]. Disruption of the pRb-E2F complex releases free E2F, which results in E2F-induced transcription leading to upregulation of CDK2 and cyclins A and E. This activity of E7 is considered critical for driving cell cycle progression in a differentiating epithelium, thus providing an environment suitable for DNA replication.

In addition to the pRb interaction, E7 also binds to the other pocket proteins p107 and p130. These play important roles in the regulation of cellular proliferation, differentiation and apoptosis, through their interactions with different molecules [127]. They inhibit E2F-mediated transcription and negatively regulate the transitions from G0 to G1, and into the S phase of the cell cycle [128], and the integrity of the pocket proteins is also required for controlling the permanent exit from the cell cycle in G2 [129]. p107 is primarily expressed in proliferating cells and inhibits E2F4 [130], while p130 is predominantly found in non-proliferating cells and inhibits E2F5 activity [131]. In addition, recent studies have shown that E7 binds and inactivates E2F6, a protein that interacts with polycomb complexes, leading to changes in epigenetic profile linked to gene silencing [132,133]. Importantly, the same LXCXE motif of the E7 CD2 domain that has been shown to be required for pRb inactivation is also required for down-regulation of p107 and p130 [134]. This again points out the necessity for the interaction of E7 with the pocket proteins for its optimal ability to continually drive cell cycle progression.

Amino acid residues 38–98 constitute the C-terminal CD3 region (Figure 3B), which contains four highly conserved cysteine residues and is involved in interactions with numerous cellular proteins, including the p21 and p27 CDK inhibitors, whose activities E7 abrogates as a necessary step in inducing cell cycle progression and overcoming DNA damage-induced cell cycle arrest; although other E7 domains are also involved in these interactions [134,135]. E7 is also involved in induction of protein kinase B (PKB) phosphorylation of p21, thereby abrogating its nuclear activities [136]. The E7 CD3 domain also binds to the Mi2β component of the histone deacetylase (HDAC) complex [137], inhibiting its activity and, leading to upregulation of the E2F gene through acetylation of the E2F promoter [138]. The E7 CD3 also binds TBP [139], and the hTid-1 protein, a member of the DnaJ-family of chaperones. The large tumor antigens of polyomaviruses, including SV40, contain J-domains that are important for viral replication, as well as for cellular transformation, and the fact that E7 interacts with a cellular DnaJ protein implies that these two oncoproteins could target common regulatory pathways via J-domains [140]. In addition to its main role in driving cell cycle progression, the interactions with substrates described above also indicate that E7 has a crucial role in destabilizing transcriptional complexes and in chromatin remodeling, consequently having an impact on cellular proliferation.

#### 3.2.2. Alpha-HPV E7 and Centrosomal Abnormalities

Centrosomes are the major microtubule organizing centers in the majority of animal and human cells [141]. Under normal conditions, each of the two centrioles that make up a G1 phase centrosome functions as a template for exactly one newly synthesized daughter centriole [142]. To prevent any possible multipolar mitoses or chromosomal instability [143,144], cells go through a single round of centriole duplication per cell division [145]. In contrast, tumor cells frequently have multiple centrosomes [146]. It is believed that aberrant centrosome numbers develop through cell division collapse or via genuine disruption of the centriole duplication cycle itself [147]. In addition, many oncogenic stimuli appear to result in the induction of abnormal centrosome and centriole numbers in vitro, but the precise mechanisms still need to be resolved.

Both E6 and E7 oncoproteins have been shown to independently cause centrosomal abnormalities when stably expressed in cell culture and in transgenic mouse models [43,148]. However, ectopic E7 expression in primary and tumor-derived cells rapidly stimulates elevated centrosomal numbers, in some cases even before the cells progress to malignancy. This might suggest that E7 has a direct effect on centrosome duplication [149], or it might occur as a consequence of E7 interacting with the p53 pathway [150], it having been shown to be pRb-independent [151]. More recent mechanistic studies have shown that E7-stimulated cyclin/CDK2 activity is crucially involved in the abnormal centrosome duplication [152]. Furthermore, it has also been reported that continuing RNA pol II-mediated gene transcription is required for HPV-16 E7-induced centriole overduplication, but not for normal centriole duplication and cell cycle progression [153].

## 4. High Risk α-HPV Infections at Different Anatomical Sites

### 4.1. Cervix

The most extensively investigated site of HPV-induced malignancy is the anogenital region, where almost all cancers arise at the cervical or anal transformation zones [154,155], where the stratified and columnar epithelia meet. It is unclear why this region is particularly susceptible to HPV-induced transformation, but it could be due to mis-regulation of viral gene expression, resulting in failure of the productive life cycle [156,157]. The cervical transformation zone contains specialized cells known as reserve cells [158] and also has a population of cuboidal cells at the squamo-columnar junction [159,160]. Depending on the environment, these cells can support either the stratified epithelium of the transformation zone or the columnar epithelium of the endocervix. In the ectocervix, a population of conventional epithelial stem cells is also present. It is thought that the individual cell type characteristics determine the fate of any given HPV infection. The pattern of viral gene expression may vary depending on the cell type, which might ultimately result in different outcomes [161]. The currently existing model argues that productive viral life cycles are likely to be completed at the ectocervix, while a collapse of the viral life cycle or incomplete infection is more likely to occur at the endocervix [154].

### 4.2. Head-and-Neck Region

Recently, HR HPV infections have also been associated with a number of head-and-neck cancers (HNSCC), the majority occurring in the oral cavity and the tonsillar crypts [162,163,164,165]. The tonsillar crypts are lined with a specialised squamous epithelium, reticulated epithelium, which is infiltrated with lymphoid tissue, fragmenting the epithelial barrier [165]. The cells here are “basaloid”, suggesting susceptibility to HPV infection, and this is also thought to be an immune-privileged zone, facilitating immune evasion by the virus. Equally importantly, this tissue does not maintain the polarization and surface maturation found in stratified squamous epithelium. Thus, this tissue may be uniquely susceptible to HPV-induced carcinogenesis, having three weaknesses: lack of barrier and basal cell type allowing easy infection; immune privilege preventing clearance; and reduced polarisation of the epithelium increasing the likelihood of a persistent infection—a major risk-factor for malignancy. The overwhelming majority of HPV-related cancers in these areas are HPV-16 positive, with a very low proportion of other HR HPV types, such as 18, 31, and 33 [4,5,166,167,168], and the reasons for this are not yet clear. Interestingly, the time between initial infection and disease development in head-and-neck cancers is much shorter than in anogenital cancers [169]. All this suggests a complex interplay between the virus and the replicative environment in this anatomical site.

About 80% of cervical cancers are caused by HPV-16 and HPV-18 and many E6 and E7 cellular targets are common to both virus types, with any differences lying only in the degree of interaction. In contrast, virtually nothing is known about their cellular substrates in the oropharynx, which may be very different owing to the tissue complexity and the surrounding environment. Biochemical and mechanistic studies of the viral oncogenes and their interacting partners in oropharyngeal cells are needed to clarify potential differences both in the productive life cycle of the virus and in virus-induced malignancy.

## 5. The Beta E6 and E7 Proteins

The E6 and E7 oncoproteins of the β-HPV types share some common interacting partners and affect similar cellular pathways as the HR α-HPV oncoproteins, but there are also some significant differences.

### 5.1. Beta-HPV E6

#### 5.1.1. Ubiquitin Ligases and p53 as Interacting Partners of Beta-HPV E6

Unlike HR α-types, the majority of the β-type E6 proteins do not target p53 for proteasome-mediated degradation [170]. However, some, such as HPV-49 E6 can target p53 for E6AP-dependent proteasome-mediated degradation [171], whereas others, such as HPV-38 and HPV-92 E6 proteins bind to p53, but in contrast to the HR α-types, they appear to stabilize p53 [119]. Most β-type E6s do not bind E6AP or bind it only weakly in vivo, which could explain, at least in part, why most of them do not target p53 for proteasome-mediated degradation. Interestingly, in vitro analyses have shown that HPV-38 E6 binds strongly to GST-E6AP, while HPV-24 E6 barely binds to GST-E6AP, yet co-expression of E6AP in cells appears to stabilize the E6 proteins of a number of β-HPV types, type 24 included. This suggests that additional proteins or protein modifications may contribute to HPV-24 E6 binding to E6AP [172]. In addition, HPV-38 E6 interacts with the ubiquitin ligase p600/UBR4, but the consequences of this interaction are still unclear [172].

#### 5.1.2. Beta-HPV E6 and NOTCH Signaling

β-type E6 proteins interact very strongly with MAML1, through a binding motif similar to the LXXLL motif used by α-type E6s to bind E6AP [119,173]. MAML1 is a transcriptional co-activator of Notch-regulated genes and the binding of β-HPV E6s proteins inhibits Notch signaling; at least in the case of HPV-8 E6, by inhibiting RBPJ/MAML1 transactivation complexes. Since Notch inhibition slows epithelial differentiation, this might affect viral replication and could also contribute to viral oncogenesis [174]. Interestingly, α-type E6s preferentially interact with E6AP, while the majority of the β-type E6s were shown to interact with MAML1 [119]. It is likely that these differences in binding preferences were evolutionary adaptations of the viruses to the differences in the tissue in which they replicate. Moreover, E6 targeting MAML1 could have been a crucial adaptation, allowing these viruses to replicate in cutaneous epithelia, since in keratinocytes Notch signaling supports cellular differentiation and growth arrest [175,176].

#### 5.1.3. Beta-HPV E6 and p53 Gene Regulation

Like α-HPV types, β-types also interfere with the expression of p53-regulated genes by associating with the transcriptional co-activators CBP and p300. The E6s from HPV-5, HPV-8 and HPV-38 all interact with p300 [16,177], and HPV-8 E6 was also shown to induce p300 degradation [16], dramatically reducing the levels of ATR protein, which plays a critical role in UV-induced damage signaling. This results in an increase in thymidine dimer mutations upon exposure to UV irradiation [16]. The HPV-38 E6 interaction with p300, on the other hand, blocks p53 acetylation and inhibition of its transcriptional functions [177]. In addition, HPV-23 E6 can block UV-induced p53 phosphorylation at residue serine 46, via its association and inhibition of HIPK2 kinase [178].

#### 5.1.4. Beta-HPV E6 and Telomerase

The E6 proteins of β-HPV types 5, 20, 22 and 38 also associate with and activate hTERT in an E6AP-dependent manner, albeit to a lesser extent than HPV-16 E6, but the E6 proteins from other β-types show a significantly lower ability to activate telomerase [179]. Furthermore, the intensity of the telomerase activation appears to be directly dependent on the strength of association between E6 and E6AP or NFX-91. Therefore, HPV-38 E6, which interacts strongly with E6AP was shown to efficiently immortalize primary HFKs in cooperation with E7 [180,181].

#### 5.1.5. Beta-HPV E6 and Apoptosis

Like the α-type E6 proteins, the β-HPV E6s can abolish p53-dependent and -independent apoptosis, which is induced in cutaneous sites in response to UV-induced DNA damage [70]. β-types can also inhibit the pro-apoptotic protein Bak and drive its proteasomal degradation; this process is also E6AP-dependent. Recently it has been suggested that the HERC1 ubiquitin ligase is required for HPV-5 E6-mediated Bak degradation [182]. Bak proteins levels are undetectable in HPV-positive skin cancers, in contrast to HPV-negative cancers, which express Bak, and which presumably abrogate the apoptotic response at a different point in the pathway [70,71,183]. Thus, Bak is one of the few highly conserved targets of both α- and β-type HPV E6 proteins.

### 5.2. Beta-HPV E7

Cellular interacting partners of β-HPV E7 proteins have not been as well-characterized as those of the α-type HR E7s. However, they also contain an intact LXCXE binding motif and interact with the pocket proteins pRB, p107 and p130 [184,185], but only HPV-38 E7 has yet been shown to induce pRb degradation, and then only in rodent fibroblasts: in human keratinocytes it inhibits pRb function by inducing its phosphorylation [171,180]. Another binding partner common to both α- and β-types is the UBR4/p600 ubiquitin ligase, but the significance of this interaction in the viral life cycle is still unclear [119].

In addition to its role in regulating cell cycle progression, HPV-38 E7 was shown to induce changes in p53-regulated gene expression [186,187,188]. It cooperates with E6 to induce p53 stabilization in the skin of transgenic mice, which then selectively induces transcription of the ΔNp73α isoform of p73. This, in turn, blocks p53’s transcriptional activation of genes involved in regulation of cell growth and apoptosis [186,187]. Furthermore, HPV-38 E7 was shown to directly regulate IKKβ nuclear translocation and ΔNp73α accumulation: these two, together with DNA methyltransferase 1 (DNMT1) and enhancer of zeste homolog (EZH2) form the ΔNp73α/IKKβ/DNMT1/EZH2 complex, which inhibits a number of p53-regulated promoters [188]. Additionally, immortalization of skin keratinocytes by HPV-38 E6/E7 is associated with hTERT gene overexpression, which is partly due to ΔNp73α accumulation. However, during proliferation HPV-38 E6/E7-positive keratinocytes show incomplete induction of telomerase with consequent telomere shortening, which leads to genomic instability and may contribute to cellular immortalization [181,189].

Only two β-HPV types, HPV-8 and HPV-38, have actually been shown to promote tumorigenesis in transgenic mouse models [190,191]. When either the HPV-8 early region, HPV-8 E6 or HPV-38 E6/E7 were expressed from a K14 promoter, this resulted in the formation of epidermal hyperplasia and, occasionally, squamous carcinomas. In addition, UV irradiation greatly increased the numbers of squamous cell carcinomas in these mice at levels that did not induce tumors in nontransgenic mice.

## 6. β-HPV Infections of Cutaneous Epithelia

The environment of viral infection of the β-HPV types is significantly different from the environment where HR α-types infect their host cells. Different β-HPV types infect and cause lesions in various different types of skin, commonly in proximity to hair follicles or nails, as well as between the digits and at the eccrine and apocrine sweat apparatus [154], all of which are regions where different tissue types are in close proximity. The majority of cutaneous stem cells are located in the buccal area of the hair follicles [192,193] and it is believed that the virus reaches those cells either through microinjuries or via hair follicles. Between the hair follicles, the tissue stem cells are located at the rete ridges and the dermal papilla (fingerprints) [194]. In the sweat glands, there are two distinct stem cell populations, located either in the gland or the duct, and both of which are susceptible to β-HPV infection, It has been demonstrated that these cells repair damaged skin, and HPVs are believed to gain access to these stem cell populations either via microtraumas or via the eccrine duct [154].

## 7. Conclusions

It is becoming clear that whilst there are some intriguing similarities between alpha and beta HPV types, there are also many major differences, and this is most likely a reflection of their differing sites of replication. In addition, it is also apparent that the same virus type, for example HPV16, might well display different properties depending upon the site of infection. Certainly its cancer-causing activity appears to be much higher in tissues with a transformation zone, such as the cervix, anus or nail-bed, than in those without. Thus, whilst we are gaining a greater understanding of how the viral oncoproteins function, there is still much to learn about their respective activities in different tissue types and environmental settings.
